# Climate Change Could Change Rates of Evolution

**DOI:** 10.1371/journal.pbio.1001015

**Published:** 2011-02-01

**Authors:** Liza Gross

**Affiliations:** Senior Science Writer/Editor, Public Library of Science, San Francisco, California, United States of America

**Figure:**
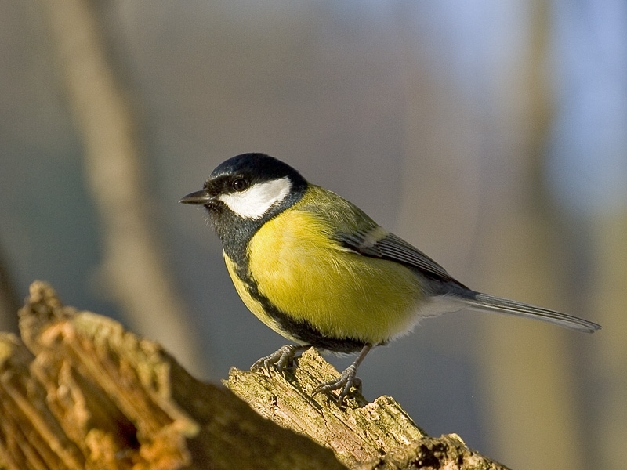
A positive link between the strength of selection and the expression of genetic variance in a wild songbird population of great tits in the Netherlands suggests that changing environmental conditions could exert a strong influence on the pace at which populations respond to selection. Image credit: Sławomir Staszczuk.

When delegates for the United Nations climate change summit met in Mexico in December 2010, they heard the latest reports of rising temperatures, rising tides, shrinking sea ice, and crashing populations of endangered species. With thousands of the planet's animals and plants facing extinction, predicting how species may—or may not—adapt to rapid changes in their environment is a top priority for biologists. Although scientists expect climate change to hasten the extinction of many species already struggling in ravaged landscapes, a new study of great tits (*Parus major*) reveals that a largely overlooked mechanism may help some wild populations adapt to climate change more quickly.

Evolutionary biologists predict a species' likely response to selection by determining the strength of selection on a trait critical to survival (for example, birth weight or age of reproduction) and its underlying genes. The strength of selection (that is, the difference in the trait between successful and unsuccessful parents) and the direction of selection (whether a trait disappears or spreads through a population) can change with environmental conditions. The expression of genes controlling a trait can also shift—though few researchers have explored this phenomenon—and may boost the adaptive response to a rapidly warming planet.

Two key variables determine rates of evolutionary change in wild plant and animal populations: the strength of natural selection and the amount of genetic variance available for selection to act on. The stronger the selection on a trait, the greater the difference in the expression of that trait between successful and unsuccessful parents. The higher its heritability—that is, the proportion of the variation in the trait attributed to genetic factors—the larger the magnitude of change across generations. 

Environmental conditions such as rising temperatures can affect both the way selection acts on a trait and the expression of genes controlling it. If the environmental effect strengthens selection or increases the genetic variance, then the rates of evolution should be accelerated.

To understand how changing temperatures affect both natural selection and genetic variance in traits in wildlife populations, Arild Husby, Marcel Visser, and Loeske Kruuk took advantage of a detailed long-term study of a population of songbirds, the great tit, in the Netherlands. The study population has experienced rising temperatures over the past 35 years. The authors focused on the effects of changing temperatures on the timing of breeding, or egg-laying, a critical trait for many bird populations in seasonal environments. They used egg-laying dates, which had been determined from recorded visits to nesting boxes during the breeding season (from more than 3,800 breeding records for nearly 2,400 females), and average daily temperatures calculated from national weather service data.

Consistent with previous work, the authors found strong selection on laying date, with early breeding birds showing higher fitness. This selection, however, was not constant but became stronger in years with warmer spring temperatures. Using information on the relatedness of individuals in the population, the authors estimated the extent to which differences between individuals were due to genetic effects. Fitting a statistical model in which estimates of genetic variance changed with different temperatures revealed an increase in genetic variance with the warmest temperatures. And, finally, tests for an association between the strength of selection and the expression of genetic variance in laying date revealed higher estimates of genetic variance when selection was strong.

The findings of a positive link between the strength of selection and the expression of genetic variance, the authors argue, suggest that changing environmental conditions could exert a strong influence on the rates at which populations respond to selection. The case of the great tits suggests that rising temperatures could potentially accelerate rates of evolutionary response. Yet despite its apparent potential for faster rates of adaptation, this population has declined over the decades studied—probably because the timing of reproduction is no longer in sync with the peak abundance of their caterpillar diet.

As climate change threatens to hasten the extinction of many of the world's vulnerable species, this study provides a clear indication that it also has the potential to affect the pace of evolution in wildlife populations. Whether this mechanism can ultimately help the great tits or other populations weather a rapidly warming world remains unclear.

**Husby A, Visser ME, Kruuk LEB (2011) Speeding up Microevolution: The Effects of increasing Temperature on Selection and Genetic Variance in a Wild Bird Population. doi:10.1371/journal.pbio.1000585**

